# A *CLAVATA3*-like Gene Acts as a Gynoecium Suppression Function in White Campion

**DOI:** 10.1093/molbev/msac195

**Published:** 2022-09-27

**Authors:** Yusuke Kazama, Moe Kitoh, Taiki Kobayashi, Kotaro Ishii, Marc Krasovec, Yasuo Yasui, Tomoko Abe, Shigeyuki Kawano, Dmitry A Filatov

**Affiliations:** Graduate School of Bioscience and Biotechnology, Fukui Prefectural University, 4-1-1 Kenjojima, Matsuoka, Eiheiji-cho, Japan; RIKEN Nishina Center, 2-1 Hirosawa, Wako, Saitama 351-0198, Japan; Graduate School of Bioscience and Biotechnology, Fukui Prefectural University, 4-1-1 Kenjojima, Matsuoka, Eiheiji-cho, Japan; Graduate School of Bioscience and Biotechnology, Fukui Prefectural University, 4-1-1 Kenjojima, Matsuoka, Eiheiji-cho, Japan; RIKEN Nishina Center, 2-1 Hirosawa, Wako, Saitama 351-0198, Japan; National Institutes for Quantum and Radiological Science and Technology, 4-9-1 Anagawa, Inage-ku, Chiba 263-8555, Japan; Department of Plant Sciences, University of Oxford, Oxford OX1 3RB, UK; Sorbonne Université, CNRS, UMR 7232 Biologie Intégrative des Organismes Marins (BIOM), Observatoire Océanologique, 66650 Banyuls-sur-Mer, France; Graduate School of Agriculture, Kyoto University, Kitashirakawa Oiwake-cho, Sakyo-ku, Kyoto 606-8502, Japan; RIKEN Nishina Center, 2-1 Hirosawa, Wako, Saitama 351-0198, Japan; Department of Integrated Biosciences, Graduate School of Frontier Sciences, The University of Tokyo, Kashiwa, FSB-601, 5-1-5 Kashiwanoha, Kashiwa, Chiba 277-8562, Japan; Future Center Initiative, The University of Tokyo, 178-4-4 Wakashiba, Kashiwa, Chiba 277-0871, Japan; Department of Plant Sciences, University of Oxford, Oxford OX1 3RB, UK

**Keywords:** sex determination, dioecy evolution, gynoecium suppression, white campion, *CLV3*

## Abstract

How do separate sexes originate and evolve? Plants provide many opportunities to address this question as they have diverse mating systems and separate sexes (dioecy) that evolved many times independently. The classic “two-factor” model for evolution of separate sexes proposes that males and females can evolve from hermaphrodites via the spread of male and female sterility mutations that turn hermaphrodites into females and males, respectively. This widely accepted model was inspired by early genetic work in dioecious white campion (*Silene latifolia*) that revealed the presence of two sex-determining factors on the Y-chromosome, though the actual genes remained unknown. Here, we report identification and functional analysis of the putative sex-determining gene in *S. latifolia*, corresponding to the gynoecium suppression factor (*GSF*). We demonstrate that *GSF* likely corresponds to a Y-linked *CLV3*-like gene that is specifically expressed in early male flower buds and encodes the protein that suppresses gynoecium development in *S. latifolia*. Interestingly, *GSFY* has a dysfunctional X-linked homolog (*GSFX*) and their synonymous divergence (d*S* = 17.9%) is consistent with the age of sex chromosomes in this species. We propose that female development in *S. latifolia* is controlled via the WUSCHEL-CLAVATA feedback loop, with the X-linked *WUSCHEL*-like and Y-linked *CLV3*-like genes, respectively. Evolution of dioecy in the *S. latifolia* ancestor likely involved inclusion of ancestral *GSFY* into the nonrecombining region on the nascent Y-chromosome and *GSFX* loss of function, which resulted in disbalance of the WUSCHEL-CLAVATA feedback loop between the sexes and ensured gynoecium suppression in males.

## Introduction

Most animal species are comprised of separate male and female individuals, which raise the question: how do separate sexes originate and evolve? Plants provide an excellent opportunity to address this question as separate sexes (dioecy) evolved many times independently and quite recently in different genera (Renner 2014; Charlesworth 2019). Evolution of separate sexes and the diversity of plant mating systems stimulated significant research efforts to understand the drivers of mating system evolution in plants (Charlesworth and Charlesworth 1978; Lloyd 1979; Renner and Ricklefs 1995; Barrett 2010; Yakimowski and Barrett 2016). The evolution of dioecy from hermaphroditism provides self-pollination avoidance mechanisms and helps to balance the investments into male and female functions (Charlesworth and Charlesworth 1981; Aonuma et al. 2021). It is thought that there are two main pathways to evolve dioecy from the ancestral hermaphroditic state: (1) via gynodioecy, when a population consists of females and hermaphrodites and (2) via monoecy, when a plant has separate male and female flowers on the same individual (Lloyd 1980; Dorken and Barrett 2004). The monoecy scenario is thought to be limited to monoecious plants (Lloyd 1980; Renner and Ricklefs 1995; Renner 2016), where flowers are already unisexual, though the relative importance of these two pathways remains unclear (Bawa 1980; Renner and Ricklefs 1995; Weiblen et al. 2000; Spigler and Ashman 2012; Renner 2016). This paper focuses on the gynodioecy pathway that is thought to have been followed by many of the dioecious plants (Dufaÿ et al. 2014).

Many of the species where sex determination and/or sex chromosomes were studied belong to primarily dioecious clades where initial transition to dioecy likely occurred a very long time ago. For example, persimmon (Akagi et al. 2014), papaya (Aryal and Ming 2014), hop (Skof et al. 2012), *Cannabis* (Van Bakel et al. 2011), *Coccinia* (Sousa et al. 2016), *Mercurialis* (Obbard et al. 2006), *Populus* (Geraldes et al. 2015) all belong to ancestrally dioecious clades (Renner 2014). Although some species in such ancestrally dioecious plant groups may reverse to a cosexual state and reevolve dioecy secondarily, such secondary transitions to dioecy may mis-inform us about the processes accompanying initial evolution of separate sexes. For example, the genomes of nondioecious species in ancestrally dioecious plant genera may be “preadapted” to sexual dimorphism, with regulatory pathways for sexually dimorphic gene expression already in place, minimizing sexual conflict upon secondary transition to dioecy. *Silene* (Bergero et al. 2015; Papadopulos et al. 2015; Kazama et al. 2016; Qiu et al. 2016) and *Rumex* (Hough et al. 2014) represent good examples of plant genera with relatively recently evolved derived dioecy, but the molecular bases of sex determination have not been dissected in these genera.

The evolution of dioecy via gynodioecy is thought to involve the spread of at least two separate mutations—a male and a female sterility mutations (Charlesworth and Charlesworth 1978; Charlesworth 2019). The spread (but not fixation) of a male sterility mutation turns a cosexual population into a gynodioecious one (hermaphrodites + male-sterile females), whereas the spread of a female sterility mutation turns hermaphrodites into males and gynodioecy into dioecy. Then, suppression of recombination between male and female sterility mutations to prevent their presence in the same individual (which would be a neuter) is thought to give rise to sex chromosomes (Charlesworth 2015). Although most studied dioecious plants contain only small (∼1 Mb) sex-determining (SD) regions (Obbard et al. 2006; Akagi et al. 2014; Aryal and Ming 2014; Geraldes et al. 2015; Akagi et al. 2019; Veltsos et al. 2019), some species (Westergaard 1958; Charlesworth 2016), such as white campion (*Silene latifolia*), have evolved large cytologically distinguishable (heteromorphic) X- and Y-chromosomes similar to that in animals such as mammals or *Drosophila*. The lack of recombination on the sex-specific chromosome (Y- or W-chromosomes) leads to the loss of genes and the accumulation of transposable elements, resulting in genetic degeneration of Y- or W-chromosomes (Charlesworth and Charlesworth 2000, 2005; Bachtrog 2013). In accordance with this scenario, in *S. latifolia*, which has been estimated to have evolved dioecy and sex chromosomes ∼11 Ma (Krasovec et al. 2018), 45% of Y-linked genes are now not expressed, and 23% have premature stop codons (Papadopulos et al. 2015).

The classic “two-factors” (responsible for male and female sterility) scenario received a lot of attention in the literature (Weiblen et al. 2000; Charlesworth and David 2004; Dorken and Barrett 2004; Renner 2016) and was supported by studies in dioecious white campion (Westergaard 1958), asparagus (Harkess et al. 2020), and kiwi fruit (Akagi et al. 2018; Akagi et al. 2019; Müller et al. 2020; Xue et al. 2020), though the dissection of the molecular basis of sex determination in persimmon (Akagi et al. 2014), poplar (Müller et al. 2020; Xue et al. 2020), and melon (Boualem et al. 2015) appear to contradict the “two-factor” model (Ma and Pannell 2016; Renner 2016). The work on genetics of sex determination and sex chromosomes in *S. latifolia* has played central role in the development of ideas in this research field (Kejnovsky and Vyskot 2010; Charlesworth 2018). Classic cytogenetic and genetic work in *S. latifolia* has identified two regions on the Y-chromosome containing sex-determining genes: the gynoecium suppressing factor and the stamen promoting factor (Westergaard 1946; Farbos et al. 1999; Lardon et al. 1999). The third Y-linked region responsible for anther maturation was also identified (Westergaard 1946). The actual genes corresponding to these sex-determining factors remain unknown and the search for the sex-determining genes has been difficult due to the large size of the genome (3 Gb) and the Y-chromosome, which is estimated to be 570 Mb (Široký et al. 2001), with a large nonrecombining region (Bergero et al. 2013; Krasovec et al. 2020). This paper is the first to identify and describe a likely sex-determining gene in *S. latifolia*. We demonstrate that this gene corresponds to the Y-linked gynoecium suppression factor (*GSF*).

## Results

### A Sex-determining Gene *GSF* Encodes a *CLAVATA3* Homolog

**Fig. 1.**
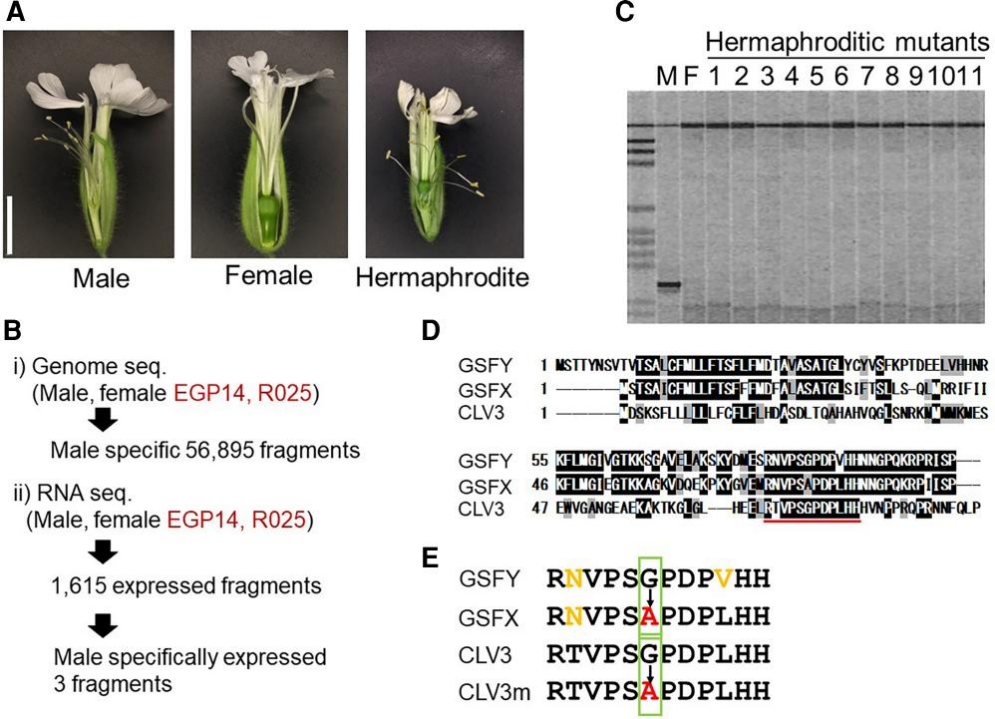
Isolation of *S. latifolia GSF* gene and its homology to *A. thaliana CLV3* gene. (*A*) Photographs of flowers of the male, female, and hermaphroditic mutant *S. latifolia* plants. Bar 1 cm. (*B*) Scheme of the *GSFY* gene isolation. (*C*) Genomic PCR analysis of the *GSF* candidate in male, female, and 11 hermaphroditic mutants (EGP4, EGP5, EGP6, EGP8, EGP9, EGP10, EGP11, EGP12, EGP13, EGP14, and EGP15). All mutants showed no amplification by the candidate specific primers. (*D*) Deduced amino acid sequence homology between Y-linked *GSF* (*GSFY*), X-linked *GSF* (*GSFX*), and *A. thaliana CLV3* genes. The conserved CLE domain is underlined. (*E*) Homology of deduced CLE domain sequences between *GSFY*, *GSFX*, *CLV3*, and its null allele (*clv3-1* or *clv3-5*); *CLV3m*.

To find the *GSF* region on the *S. latifolia* Y-chromosome, we used previously generated (Papadopulos et al. 2015) Illumina genomic sequence data of a male and a female from the highly inbred *S. latifolia* K-line (Kazama et al. 2003), as well as hermaphroditic Y-deletion mutants (fig. 1*A*) EGP14 and R025 from that inbred line. These two mutants were previously characterized as having the smallest deletion around the *GSF* region on the Y-chromosome (Kazama et al. 2016). EGP14 genome has been sequenced previously (Krasovec et al. 2019), whereas R025 was sequenced as part of this study (accession nos. DRR357638 and DRR357639). These sequence data were analyzed by using the k-mer subtraction approach described by Akagi et al. (2014) to identify the sequence reads and assemble genomic contigs corresponding to the Y-specific region deleted in the EGP14 and R025 mutants (see Materials and Methods). Henceforth we will refer to this region as “Ydel.”

We used the contigs from the Ydel region to blast-search for homologous expressed genes in *S. latifolia* male transcriptomes published previously (Papadopulos et al. 2015) and newly assembled in this study with Trinity and Drap (Grabherr et al. 2011; Cabau et al. 2017) (accession nos. ICSN01000001–ICSN01003571 and ICSO01000001–ICSO01092457). This identified 1,615 Ydel region sequences that had high identity (*e*-value < 0.0000001) to the expressed fragments. Mapping RNA-seq reads to these 1,615 expressed Ydel region sequences using RSEM (Li and Dewey 2011) revealed that only three genic fragments (supplementary table S1, Supplementary Material online) were expressed in male flower buds, but not in the female or the deletion mutants (fig. 1*B*). Genomic polymerase chain reaction (PCR) in male, female, and 11 hermaphroditic mutants showed that only one of these fragments was male-specific (fig. 1*C*), indicating that this gene is a strong candidate for being the sex-determining gene. Blast-X search of this fragment showed homology to an *Arabidopsis thaliana* protein, CLAVATA3 (CLV3). The *CLV3* gene is well known as a gene that controls the size of the shoot apical meristem and the flower meristem. In the *A. thaliana clv3* mutant, not only the size of SAM but also the size of flower meristem was reduced (Fletcher et al. 1999). Therefore, we assumed that the *CLV3* homolog was a strong candidate for the *GSF* gene in *S. latifolia* and we named it *GSFY* to denote its location on the Y-chromosome.

To find the homologs of the *GSFY* gene outside of the Y-chromosome we used blastn to search the female *S. latifolia* genome (Papadopulos et al. 2015), which revealed a homolog with 87% identity to the *GSFY*. This blast hit was located only 11 kb away from the gene “contig2443” genetically mapped to the X-chromosome in the previous study (Papadopulos et al. 2015). X-linkage of this genomic region was also confirmed using segregation analysis with genomic sequence data from *S. latifolia* parents and F1 progeny (Krasovec et al. 2018). Further blastn search against a female *S. latifolia* transcriptome (Krasovec et al. 2019) identified corresponding cDNA 288 nucleotides long. Henceforth, we will refer to this gene as *GSFX*. An alignment of the deduced amino acid sequence showed that both *GSFY* and *GSFX* were homologous to *A. thaliana* CLV3, especially in the conserved CLE domain (fig. 1*D*). The *GSFX* gene is likely dysfunctional because it contains the same mutation as the *A. thaliana clv3-1* and *clv3-5* mutants, which causes a single amino acid change (from Gly to Ala) in the CLE domain (fig. 1*E*). This amino acid change is sufficient to render CLV3 dysfunctional (Fletcher et al. 1999). On the other hand, this Gly residue was conserved in the CLE domains of the *GSFY* and *CLV3* of *A. thaliana*. These results suggest that *GSFY* has a function in controlling the size of the flower meristem, but *GSFX* does not.

### *GSFY* is Expressed in the L3 Layers of Small Flower Buds

**Fig. 2.**
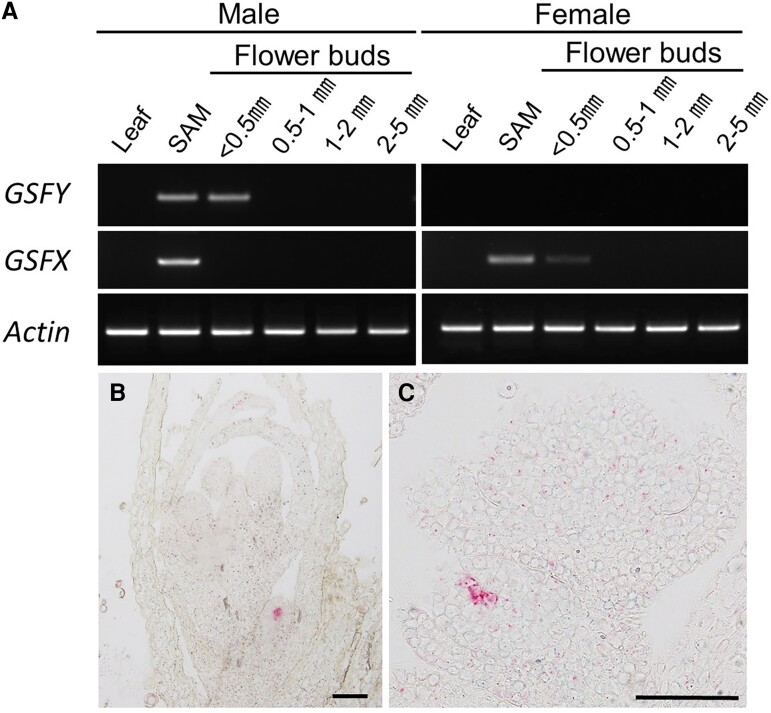
Expression of *GSFY* and *GSFX* genes. (*A*) RT-PCR for *GSFY* and *GSFX* on leaf, SAM, and flower buds at different developmental stages (lengths of flower buds are indicated). (*B* and *C*) *In situ* hybridization images for *GSFY* in developing flower buds. Red signals derived from *GSFY* probe were detected at stages 1–2 of the developing flower buds, but not at progressed stages. Bars, 100 μm.

The expression of both *GSFY* and *GSFX* was examined by RT-PCR, which revealed that both *GSFY* and *GSFX* were expressed in shoot apical meristem, but only *GSFY* was expressed in small (<0.5 mm) flower buds at the early stages of their development (fig. 2*A*). To investigate the expression pattern of both genes, RNA *in situ* hybridization to tissues using BaseScope kit was performed on developmental flower buds (See Materials and Methods). No hybridization was detected for the *GSFX* probe, whereas for the *GSFY* probe hybridization signals were detected in the L3 layers of the small flower buds at stages 1–2 (fig. 2*B* and *C*). This pattern of *GSFY* hybridization is consistent with the function of this gene to suppress gynoecium development at early stages of development in male flowers. In *A. thaliana*, gynoecium primordia originate from L3 layers (Jenik and Irish 2000). Thus, *GSFY* product may work in L3 layers to repress gynoecium development in *S. latifolia*.

### *GSFY* is Functional But *GSFX* is Not

**Fig. 3.**
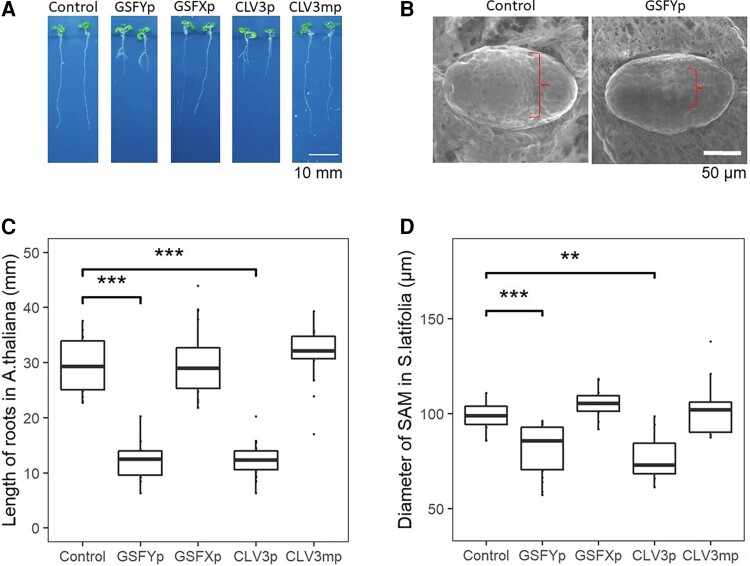
The bioassay of CLE peptides. (*A* and *C*) CLE peptide treatments on *A. thaliana* seedlings to investigate their effects on root growth. The roots were measured after 8 days of growth on solid 1/2 MS media with 0.1 μM of different peptides (GSFYp, GSFXp, CLV3p, and CLV3mp). (*A*) Photographs of two different plants per treatment. (*C*) Quantitative measurements of the root length (*n* = 18 for each treatment). GSFYp and CLV3p showed significant defect on the root growth (*P* < 0.01, Wilcoxon rank-sum test), whereas GSFXp and CLV3mp showed almost no effect. (*B* and *D*) CLE peptide treatments on *S. latifolia* seedlings to investigate their effects on SAM size. The SAMs were observed by SEM after 14 days of growth on liquid 1/2 MS media with 0.1 μM different peptides followed by calculation of SAM diameter by ImageJ. (*B*) Photographs of representative samples. (*D*) Quantitative measuring of the SAM diameter (*n* = 18 for each treatment). Similar to the *A. thaliana* experiment, GSFYp and CLV3p showed significant defects of the SAM size (*P* < 0.01, Wilcoxon rank-sum test), whereas GSFXp and CLV3mp showed almost no effect. Box plots, 25th–75th percentile; center line, median; whiskers, full data range in *B* and *D*.

*Arabidopsis thaliana* CLV3 acts as a small peptide, so the function of the CLV3 variants can be analyzed by a bio assay in which Arabidopsis plants are treated with synthetic peptides (Fiers et al. 2005; Kondo et al. 2006). The peptides encoded by *CLV3*, the mutated variant of *CLV3* found in *clv3-1* and *clv-3-5* mutants, *GSFY*, and *GSFX* were synthesized with replacements of proline residues by hydroxyproline residues, as described previously (Kondo et al. 2006). To investigate the activity of the peptides encoded by *GSFY* (GSFYp), *GSFX* (GSFXp), as well as the peptides encoded by *CLV3* (CLV3p) and by the mutated *CLV3* (CLV3mp), these peptides were synthesized and used to treat *A. thaliana*. Root elongation was significantly inhibited in the CLV3p and GSFYp treatments (*P* < 0.01, Wilcoxon rank-sum test), but not at all in the CLV3mp and GSFXp treatments (*P* > 0.05, Wilcoxon rank-sum test) (fig. 3*A* and *C*). The root length after CLV3mp and GSFXp treatments was comparable to that of the nontreated col-0 (fig. 3*A*). This tendency was also confirmed on the SAM in *A. thaliana* (supplementary fig. S1, Supplementary Material online). The function of these peptides was also tested in the SAM of *S. latifolia.* Similar to the bioassay in *A. thaliana*, the CLV3p and GSFYp treatments significantly decreased the diameter of the *S. latifolia* SAM in both sexes (*P* < 0.01, Wilcoxon rank-sum test), whereas the CLV3mp and GSFXp treatments showed almost no effect on the SAM size (*P* > 0.05, Wilcoxon rank-sum test) (fig. 3*B* and *D*). These results indicate that the function of *GSFY* is similar to *CLV3*, whereas *GSFX* gene is likely dysfunctional.

### *GSFY* Supresses Growth of Gynoecium

**Fig. 4.**
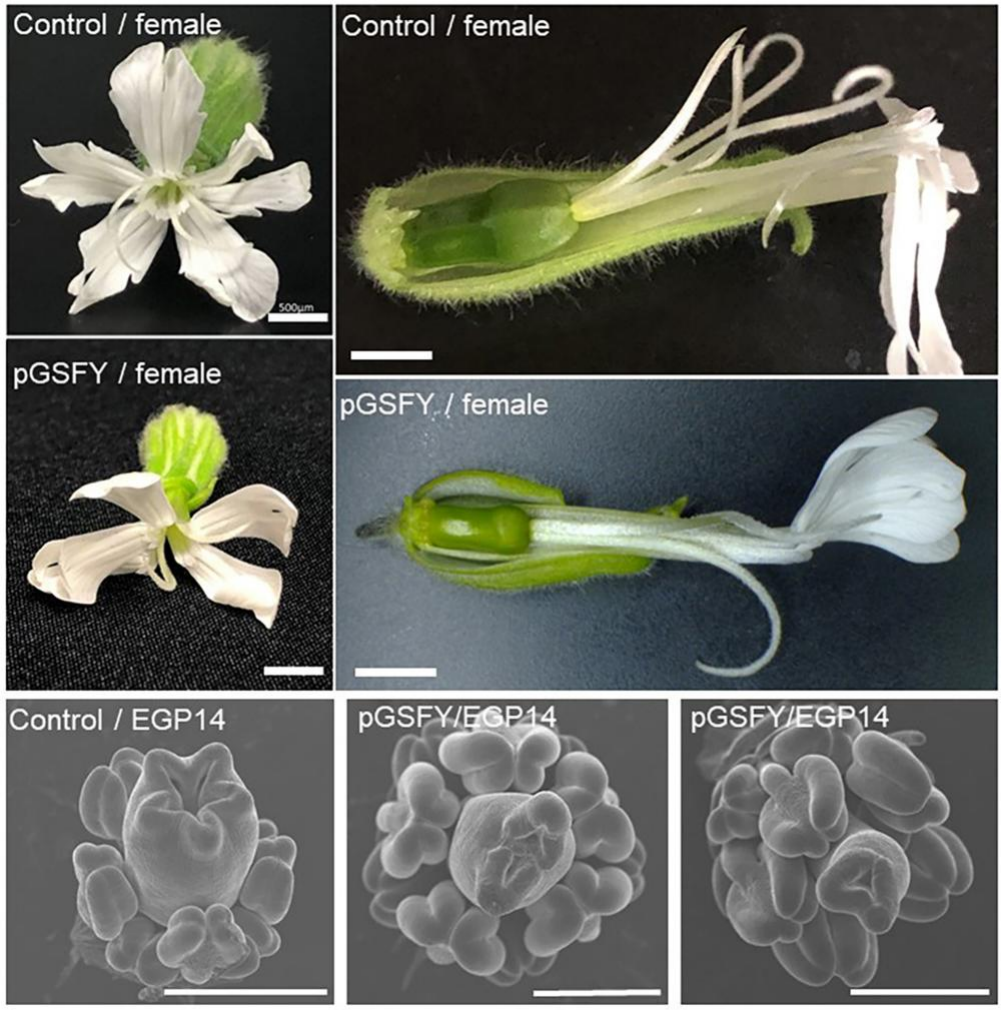
The effect of GSFYp treatment on gynoecium development in *S. latifolia* female and hermaphroditic mutant plants. Flower buds at stages 1–3 were treated with water containing 5 μM GSFYp. Open flowers were observed under stereo microscopy. GSFYp treatment decreased the size of ovaries and the number of styles in both female and hermaphrodite mutants. Bars, 500 μm.

To test the gynoecium suppressing function of *GSFY*, we performed the GSFYp treatment on the flower buds of female and hermaphroditic mutants of *S. latifolia*. After twice dropping of 0.5-µM GSFYp on the developing female flower bud, a female flower having two styles with a small ovary opened (fig. 4). The number of carpels also decreased both in females and hermaphrodites (fig. 4 and supplementary fig. S2, Supplementary Material online). These phenotypes were not observed after the treatment of GSFXp.

**Fig. 5.**
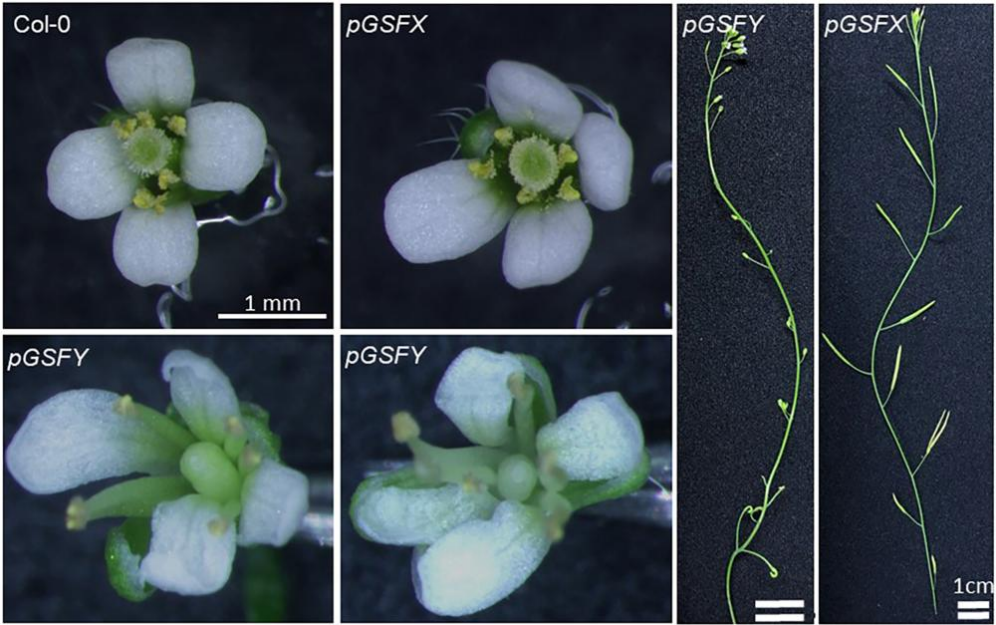
Transformation of *GSFY* and *GSFX* in *A. thaliana*. *GSFY* and *GSFX* sequences under their native promoters (*pGSFY* and *pGSFX*) were introduced into the Col-0 plants. In the T2 generation, the *pGSFY* plants showed inhibition of the pistil growth and sterility, whereas the phenotype of the *pGSFX*-transgenic T2 plants was not affected.

One of the ways to prove the function of *GSFY* in *S. latifolia* is transformation. As transformation of *S. latifolia* is problematic, we performed transformation of *A. thaliana* instead. The coding sequences of *GSFY* and *GSFX* under control of their native promoters were introduced into *A. thaliana*. The resulting transgenic line for *GSFY* showed inhibition of pistil growth and sterility in the T_2_ generation (fig. 5). Such a phenotype was not observed in the *GSFX*-transgenic plants (fig. 5). Taken together, we concluded that the Y-linked *CLV3* homolog, the *GSFY* but not the *GSFX* has a gynoecium suppressing function.

### *GSFY* Emerged When the Sex Chromosomes Evolved

Synonymous divergence between the *GSFX* and *GSFY* genes is d*S_x_*_:*y*_ = 0.18, which is higher than the average d*S_x_*_:*y*_ = 0.11 for the older stratum of *S. latifolia* sex chromosomes (Krasovec et al. 2018), but is similar to d*S_x_*_:*y*_ ∼ 0.15–0.20 reported for some of the sex-lined genes in this species, such as *SlX4/Y4* (Atanassov et al. 2001) and the genes in the upper range of the d*S_x_*_:*y*_ distribution in the older stratum (Papadopulos et al. 2015). Assuming a mutation rate in *S. latifolia* is *m* = 7.31 × 10^−9^ per nucleotide per generation (Krasovec et al. 2018), d*S_x_*_:*y*_ = 0.18 corresponds to divergence time between the *GSFX* and *GSFY* genes *T*_generations_ = d*S_x_*_:*y*_/2 m ∼ 12 million generations or *T*_years_ ∼ 18 My, assuming 1.5 years per generation on average (Richards et al. 2003). This estimate of the time since the divergence between the *GSFX* and *GSFY* genes is close to the previously estimated upper limit for the age of sex chromosomes in this species (95% CI: 7.83–15.03 My) (Krasovec et al. 2018), which is consistent with the *GSFY* gene being the sex-determining gene that evolved its sex-determining function prior to or at the same time as the cessation of recombination in the older stratum of the *S. latifolia* sex chromosomes.

In order to establish the ancestral state of the *GSF* gene before divergence into the X- and Y-linked gametologs, we identified the homologs of this gene in a nondioecious outgroup—*Silene uniflora*. For this purpose, we blast-searched a draft assembly of the *S. uniflora* genome (Papadopulos et al. 2021) with the CDS of the *GSFY* gene and found two homologs with 93% identity to *GSFY*, on contigs JAGPOY010206798.1 and JAGPOY010140724.1. The sequences of the two *S. uniflora* homologs are very similar to each other with only three nucleotide differences (in an alignment 197 nucleotides long) and apparently represent allelic variation in a single gene or a recent duplication in *S. uniflora*. The predicted protein sequence of the *S. uniflora* genes in the conserved CLE domain is the same as that in the *GSFY* and *A. thaliana CLV3*, indicating that the Gly to Ala mutation in that domain in the *GSFX* is a derived state. If the *GSFX* were dysfunctional, we would expect this gene to accumulate amino acid replacements because purifying selection should not eliminate them in a dysfunctional gene. Indeed, *GSFX* contains 12 amino acid replacements specific to this gene, whereas *GSFY* contains only three such replacements (*P* = 0.02 with Tajima’s relative rate test for amino acids). Accordingly, the nonsynonymous to synonymous substitution rate ratio (d*N*/d*S*) is much higher for the *S. latifolia GSFX* (d*N*/d*S* = 0.38) compared with the *GSFY* (d*N*/d*S* = 0.08) and the homologs in *S. uniflora* (d*N*/d*S* = 0.09). Thus, multiple lines of evidence indicate that *GSFX* is dysfunctional and it must have lost its function at least a few million years ago to allow enough time to accumulate multiple amino acid replacements.

## Discussion

Here, we report the identification and functional analysis of the gene likely responsible for the sex-determining gynoecium suppressing function (GSF) in *S. latifolia*—the dioecious plant species that inspired the development of the canonical “two-factor” model of dioecy evolution (Westergaard 1953, 1958; Charlesworth and Charlesworth 1978). This model includes the spread of a male and a female sterility mutations that lead to evolution of gynodioecy and dioecy, respectively, from the ancestral hermaphroditic state (Charlesworth 2015). The putative sex-determining gene, *GSFY*, identified in this study corresponds to the second step—the loss of female function caused by the GSF identified genetically many decades ago (Westergaard 1946, 1958). We report that the GSF likely corresponds to the Y-linked *CLV3*-like gene that is specifically expressed in early male flower buds and encodes the protein that suppresses gynoecium development in *S. latifolia* and *A. thaliana*.

According to the canonical two-factor model, the evolution of dioecy and XY sex chromosomes involves a recessive loss-of-function male sterility mutation on the proto-X and a dominant gain-of-function female sterility mutation on the proto-Y-chromosome (Charlesworth 2002). Thus, our finding of an X-linked loss-of-function mutation in *GSFX* may seem surprising and would be expected for the other sex-determining gene (*SPF* that remains to be identified in *S. latifolia*), rather than for the *GSF* studied in this paper. However, as argued below, the loss-of-function mutation in the *GSFX* gene may be an important step in dioecy evolution, contributing to the control of gynoecium suppression via the WUSCHEL-CLAVATA feedback loop (Schoof et al. 2000). Sex determination in plants is often unstable, condition-dependent and can be affected by hormone treatments (Khryanin 2002; Masuda et al. 2022). Contrary to this, sex determination in *S. latifolia* is very stable and completely genetically determined (Heslop-Harrison 1963), with plant hormone treatments having no effect on the sex expression (Ruddat et al. 1991). Recently, androhermaphrodite *S. latifolia* plants were reported to be produced by the treatment of chemicals affecting epigenetic states (Bacovsky et al. 2022). This is consistent with the fact that WUSCHEL-CLAVATA pathway is not affected by major plant hormones except for the stabilization of WUS proteins by cytokinins (Snipes et al. 2018), but controlled by epigenetic modifications (Cao et al. 2015; Shang et al. 2021).

Within the WUSCHEL-CLAVATA feedback loop, the *CLV3* mRNA production is activated by WUS protein, whereas the expression of *WUS* is repressed by *CLV3.* Expression of *CLV3* is also repressed by the WUS protein when its concentration is high (Perales et al. 2016; Plong et al. 2021). WUSCHEL plays a role in pistil growth and the *A. thaliana wus* mutant has a smaller floral meristem, frequently resulting in the absence of pistil (Laux et al. 1996). CLV3 inhibits expression of *WUS* and the *A. thaliana clv3* mutant shows an enlarged pistil (Fletcher et al. 1999). In *A. thaliana*, the termination of floral meristem is induced by the cessation of *WUS* expression followed by shutting down of *CLV3* expression, to produce gynoecium primordia (Laux et al. 1996; Mayer et al. 1998; Shang et al. 2021). Thus, shifting the balance between *WUS* and *CLV3* in the termination of floral meristem, it is possible to ensure suppression of gynoecium development in males and active carpel development in females. Importantly, the *GSFY*-transgene in Arabidopsis plants specifically affected gynoecium suppression (fig. 5). This may be due to the change of expression pattern of *GSFY*; it was expressed at the L3 layer of the male floral meristems (fig. 2*B* and *C*), whereas *CLV3* is expressed in the L1 and L2 layers of the Arabidopsis floral meristems (Brand et al. 2000). The loss of function mutation in *GSFX* also contributes to the masculinization of the male flower, limiting *GSF* expression to males and ensuring active carpel development in females. Reducing the number of copies of the *WUSCHEL* gene in males was likely another contribution to this balance shift. In particular, as reported previously (Kazama et al. 2012), the *S. latifolia WUS* homolog *SlWUS1* is linked to the X chromosome but no Y-linked copy was found. This creates a bias in the number of the *SlWUS1* gene copies between the sexes, with females having twice as many *SlWUS1* compared with males. As *SlWUS1* is not dosage compensated (Kazama et al. 2012), this copy number difference between the sexes likely leads to gynoecium enlargement in females. The results of GSFYp treatment on female and hermaphroditic plants support this hypothesis, as hermaphrodites (having one X chromosome) tended to show smaller gynoecium compared with XX-females that had two copies of *SlWUS1* gene.

Gene duplication and their sub- or neofunctionalization are expected to play a significant role in evolution of carpel suppression in dioecious plants (Ohno 1967). Consistent with this concept, genes involved in the suppression of female function, *ShyGirl* in kiwifruits (Akagi et al. 2018; Akagi et al. 2019) and *SOFF* in *Asparagus* (Harkess et al. 2017), are thought to be derived from duplications of autosomal genes. Moreover, the sex-determining gene in persimmon, *OGI*, which is a small RNA repressing autosomal feminizing gene, *MeGI*, was also derived from a duplication (Akagi et al. 2014). However, our findings do not support the role of gene duplication in evolution of gynoecium suppression during dioecy evolution in *S. latifolia*. Identification of an X-linked homolog of the Y-linked carpel suppressor *GSFY* indicates that the gene ancestral to *GSFX* and *GSFY* was already present on the proto-sex chromosomes before the cessation of recombination between the X- and Y-linked alleles of that gene. Furthermore, it is likely that *SlWUS1* was also present on the proto-sex chromosomes because the homolog of this gene in nondioecious *Silene vulgaris* is located on the same chromosome as other homologs of genes sex-linked in *S. latifolia* (Kazama et al. 2012). As no Y-linked copy of the X-linked *SlWUS1* gene was found in *S. latifolia* (Kazama et al. 2012), this gene was likely lost from the Y-chromosome, whereas the X-linked copy was retained. The analysis of gene expression in males and females revealed no dosage compensation for *SlWUS1* gene (Kazama et al. 2012). Thus, the loss of the Y-linked copy of *SlWUS1* gene further contributed to the disbalance in WUSCHEL-CLAVATA feedback loop between males and females and thus it could have been an important step in dioecy evolution. Furthermore, given the well-documented functional role of CLAVATA3 and WUSCHEL in regulation of the sizes of shoot and floral meristems (Prunet et al. 2009; Somssich et al. 2016), this disbalance between sexes may play a key role in controlling the sexual dimorphism of this dioecious plant species, where male flowers are about three times smaller than female flowers and male inflorescences are bigger than female inflorescences (Gehring and Linhart 1993).

The estimated synonymous divergence between the *GSFX* and *GSFY* is consistent with the maximal estimate of the sex chromosomes age in *S. latifolia* (95% CI: 7.83–15.03 My) (Krasovec et al. 2018) and indicates that cessation of recombination between the X- and the Y-linked *GSF* copies has occurred at the very early stage of sex chromosome evolution in this species. It is possible that inclusion of the *GSFY* into the nonrecombining region on the nascent Y-chromosome (NRY) was nearly simultaneous with the mutation in the *GSFX* gene that prevents its carpel-suppressing function. We hypothesize that inclusion of the *GSFY* into the NRY and dysfunctionalization of the X-linked copy of that gene were the two key events that led to evolution of dioecy from gynodioecy in *S. latifolia* via the WUSCHEL-CLAVATA feedback loop, as described above. Although further studies will be necessary to prove the involvement of this mechanism in carpel suppression in males, our results open the door to investigate the involvement of the X chromosome in sex determination in *S. latifolia*.

## Materials and Methods

### Plant Material

The inbred *S. latifolia* line, the K line (Kazama et al. 2003), and its Y-deletion mutants EGP14, EGP15, and R025 (Kazama et al. 2016) were used in this study. Plants were grown in pots in a regulated chamber at 23°C in a 16 h light/8 h dark cycle. The leaves and flower buds were frozen in liquid nitrogen and stored at −80 °C prior to DNA and RNA extraction. The Columbia (Col-0) ecotype and the clv3-101 mutant of *A. thaliana* were used in peptide treatment assays. The clv3-101 mutant was originally obtained by the heavy-ion irradiation (Maeda et al. 2014) and kindly provided from Dr. Ali Ferjani of Tokyo Gakugei University. *Arabidopsis thaliana* seeds were grown in either soil or 0.7% (w/v) agar-containing Murashige-Skoog (MS) medium (Wako-junyaku) supplemented with 3% (w/v) sucrose and Gamborg’s B5 vitamins at 23°C on a 16 h light/8 h dark cycle.

### Genomic Sequencing

To extract the candidate gene fragments responsible for the GSF function, we sequenced the genome of the deletion mutant R025 and a female plant. In addition, we used genome sequence data from nonirradiated plants of the inbred K-line and another deletion mutant EGP14 (Krasovec et al. 2019) as well as from parents and F1 progeny published previously (Krasovec et al. 2018).

DNA for genomic sequencing was extracted from fresh leaves using the DNA Plant Easy kit (Qiagen). For high-throughput sequencing, PCR-free Illumina libraries were prepared at Beijing Genomics Institute (BGI, Shenzhen, China). These libraries were sequenced on HiSeq4000 instrument at BGI. An additional PCR-free Illumina library of genomic DNA from R025 was independently prepared at RIKEN, and sequenced on HiSeq2500 instrument at RIKEN. All sequence data generated are listed in supplementary table S2, Supplementary Material online.

### Male-specific K-mer Extraction and Assembly of Putative Ydel Contigs

After the trimming of raw reads by Trimmomatic (version 0.36) using the parameters LEADING: 20, TRAILING:20, SLIDINGWINDOW:4:28, MINLEN:40 (Bolger et al. 2014), genomic reads were processed to produce 35-bp k-mers as described previously (Akagi et al. 2014). The python script for the k-mer extraction was kindly provided by Dr. Tsuyoshi Akagi. Briefly, we generated 35-bp k-mer starting with “A” and then kept a set of k-mer with a minimum total count threshold of 10 and a maximum total count threshold of 1000 for male + female, male + R025, or male + EGP14 respectively. Then, male-specific k-mers that were absent in female, R025, and EGP14, were identified. All pair-ended reads containing at least one male-specific k-mer were retained and assembled with SOAPdenovo2 (Luo et al. 2012).

Genomic reads of male, female, R025, and EGP14 were mapped to the resulting male-specific assembly by BWA-MEM (Li 2013). After normalizing the total counts between genomic reads, mapped count ratio of female to male and EGP14 to male were calculated. To narrow down the male-specific contigs we selected the contigs with female/male ratio of <20% or an EGP14/male ratio of <40% These were used as the Ydel assembly in the following analysis.

### Identification of the Candidate *GSF* Gene

*GSF* gene is expected to be expressed at the early stages of flower development. Thus, we analyzed transcriptome sequence data from flower buds <0.5 mm in diameter of male and female plants from the K-line, as well as from the deletion mutants R025, EGP14, and EGP15. Transcripts of R025 and male were sequenced as part of this study (accession nos. DRR359968, DRR359969, and DRR360400), whereas other transcriptome data were published previously (NCBI bioproject PRJNA474609) (Krasovec et al. 2019). For transcriptome sequencing, total RNA extraction was done with the RNA Easy kit (Qiagen) and sequenced at WTCHG on Illumina HiSeq4000 with 75 bp paired-ends reads. The four sets of reads from male flower buds (accession nos. DRR359968 and DRR360400; NCBI bioproject PRJNA474609) were assembled with Trinity and Drap, respectively (Grabherr et al. 2011; Cabau et al. 2017). The resulting contigs were BLAST-searched against the male-specific assembly to identify 1,615 expressed Ydel fragments (fig. 1*B*). All RNA-seq reads were aligned to the expressed Ydel fragments using RSEM (Li and Dewey 2011) to obtain expression value of each fragment in the male, female, R025, EGP14, and EGP15. This analysis identified three fragments that were not homologous to TE and expressed in male but not in female, R025, EGP14, and EGP15. Three primer sets were designed for the three fragments (supplementary table S3, Supplementary Material online), and PCR was performed on the genomic DNAs of male, female, and 11 hermaphroditic plants (EGP4, EGP5, EGP6, EGP8, EGP9, EGP10, EGP11, EGP12, EGP13, EGP14, and EGP15) to test the male specificity of their fragments. Only one fragment was found to be amplified from male but not from the female or the deletion mutants.

### Segregation Analysis to Confirm X-linkage of *GSFX* gene

To test whether *GSFX* is X-linked we reused the sequence data from a *S. latifolia* genetic cross published previously (Krasovec et al. 2018). That data included Illumina paired end sequences of two parents and 10 F1 progenies (4 males and 6 females). Read mapping to female genome reference and SNP calling were done as described previously (Krasovec et al. 2018). X-linkage was tested by SNP segregation, checking whether the sons always inherit the maternal allele, as expected for the X-linked genetic variants.

### Expression Analyses of the *GSFX* and *GSFY*

Total RNA was extracted from SAMs, leaves, flower buds, and open flower of male and female plants, respectively, by using the RNeasy Plant Mini kit (Qiagen). cDNA synthesis from 1 µg of total RNA was performed by using Superscript IV reverse transcriptase (Invitrogen). The cDNAs were then subjected to touch down PCR (98°C for 10 sec., Annealing temperature for 15 sec., and 68°C for 2 sec.), in which the annealing temperature was reduced from 68°C by 1°C every cycle until the calculated Tm range was reached, followed by 30 cycles (98°C for 10 sec., Calculated Tm for 15 sec., and 68°C for 2 sec.) by using KOD One polymerase (TOYOBO), with primers of GSFY_F7 and C279090_F2 for *GSFY*, and GSFX_F3 and GSFX_R2 for *GSFX*, and Slactin01 and Slactin02 for the Actin gene, respectively (supplementary table S3, Supplementary Material online). For *in situ* expression analysis, BaseScope Duplex Detection Reagent Kit (ACD, Hayward, CA, USA) was used according to the manufacturer's instructions with some modifications. Probes for *GSFY* and *GSFX* were produced by ACD, respectively. Fixation and embedding of the flower buds were performed as described previously (Kazama et al. 2009). Semithin 8-μm sections of male flower buds were made by a Microm HM 340E microtome, and transferred to Fisherbrand Superfrost Plus slides (Thermo Fisher Scientific, Pittsburg, PA, U.S.A.). The sections were dried out overnight at 37°C. After baking for 60 min at 60°C, the slides were treated with xylene, and dehydrated in ethanol. The slides were then pretreated with hydrogen peroxide (provided by the BaseScope Duplex Detection Reagent Kit) for 10 min at room temperature, incubated in a target retrieval buffer (provided by the BaseScope Duplex Detection Reagent Kit) for 15 min maintained at boiling temperature (98°C to 102°C), washed by sterilized water, dehydrated in ethanol, then dried out overnight at room temperature. The slides were treated with protease III (provided by the BaseScope Duplex Detection Reagent Kit) for 30 min at 40°C in a HybEZ hybridization oven (ACD). Hybridization and detection of the signals were performed as described in the manufacturer’s protocol. Slides were imaged on an Olympus BX53 microscope (Olympus, Tokyo, Japan).

### Peptide Treatment of the *GSFX* and *GSFY*

Following the usual CLV3 production protocol, four dodecapeptides (CLV3p, CLV3mp, GSFYp, and GSFXp) were synthesized at the RIKEN Centre for Brain Science with the modification that two proline residues were replaced with hydroxyproline residues (Shinohara and Matsubayashi 2013). In the root elongation assay with peptide treatments, the surface-sterilized Col-0 seeds were sawn on 0.7% agar-containing 1/2 MS medium (Murashige and Skoog 1962) supplemented with 3% (w/v) sucrose and 0.1 µM synthetic peptides. After 8 days growing at 23 °C on a 16 h light/8 h dark cycle, the root length was measured from the base of the hypocotyl to the tip of the primary root by using the ImageJ software. The peptide treatment assay on the SAM in the *A. thaliana clv3-101* mutant was performed as described previously (Shinohara and Matsubayashi 2013, 2015). In the peptide treatment assay on the SAM in *S. latifolia*, the surface-sterilized *S. latifolia* seeds were placed in the liquid 1/2 MS medium supplemented with 1% (w/v) sucrose and 0.1 µM synthetic peptides, and incubated at 4 °C for 3 days followed by the incubation at 23 °C on a 16 h light/8 h dark cycle for 10 days. These incubations were performed in 50 ml Falcon tubes (100 seeds/tube) with 20 mL of the liquid media on the RT-50 rotator (TAITEC, Saitama, Japan). After these incubations, the SAM of the peptide-treated seedling was observed and photographed by scanning electron microscopy (SEM) with a cool stage at −20 °C (S-3000N, Hitachi, Tokyo, Japan). The SEM was operated at 4 kV. The diameter of SAM was measured by the ImageJ software.

### Peptide Treatment on *S. Latifolia* Flower Buds

By using a micro-pipet, 0.5 µM synthetic GSFYp was dropped onto female and hermaphroditic mutants (EGP15 and EGP14) at stages 1–3 determined as described previously (Grant et al. 1994; Farbos et al. 1997). After 1 day, additional 0.5 µm synthetic *GSFY* was dropped onto the same flower buds. These peptide-treated plants were grown in a growth chamber at 23 °C on a 16 h light/8 h dark cycle. After 10–14 days, the developing flower buds at stage 8 were observed and photographed by SEM with a cool stage at −20 °C (S-3000N, Hitachi).

### Transformation of *A. thaliana*

The *GSFY* fragment was amplified by RT-PCR with the primers (GSFY_cF2 and GSFY_cR2, supplementary table S3, Supplementary Material online) by using cDNA from male flower buds as a template. The resulting fragment was subcloned in the EcoRV site of the pJET2.1 vector (Thermo Fisher Scientific, Waltham, MA, USA). The upstream region was amplified by PCR with primers GSFY_gF1b and GSFY_gcR1b (supplementary table S3, Supplementary Material online), by using the genomic DNA of K-line female as a template and inserted in the *Eco*RV site of the subclone by using the In-Fusion HD Cloning Kit (TaKaRa Bio. Inc., Shiga, Japan). The resulting subclone was used as a template for PCR amplification with primers (GSFY_gF1b and GSFY_gcR1b, supplementary table S3, Supplementary Material online) to obtain the fragment including the native promoter followed by the cDNA sequence. The amplified fragment was inserted between *Hin*dIII and *Sac*I sites of the pSMAH621 binary vector, which was kindly provided by Dr. H. Ichikawa (National Institute of Agrobiological Sciences, Tsukuba, Japan), by using the In-Fusion HD Cloning Kit, to generate *pgGSFY*. For the cloning of the *GSFX* gene, its genomic sequence was amplified by PCR with primers (GSFX_gF1 and GSFX_gcR1, supplementary table S3, Supplementary Material online) by using the genomic DNA of K-line female as a template. The amplified fragment was inserted between *Hin*dIII and *Sac*I sites of the pSMAH621 binary vector by using the In-Fusion HD Cloning Kit, to generate *pgGSFX*.

These constructs were introduced into Agrobacterium tumefaciens strain C58, respectively, and the resulting bacteria strains were used to transform wild-type *A. thaliana* (Col-0) by the floral-dip method (Clough and Bent 1998). Hygromycin-resistant T_1_ plants were self-pollinated and then the phenotype of T_2_ plants were observed under stereomicroscopy.

### Sequence Divergence Analyses

The alignments of *GSFX*, *GSFY*, and its homologs from *S. uniflora* were done with *muscle* (Edgar 2004) and checked visually in *ProSeq3* (Filatov 2009). The CDS alignments from these genes were analyzed in *MEGA* (Kumar et al. 2018) to measure the pairwise synonymous and nonsynonymous divergence (Nei and Gojobori 1986) and conduct the Tajima’s relative rates test (Tajima 1993) with *S. uniflora* used as an outgroup.

### Statistical Analysis

Statistical calculations were conducted using R (v4.03). Statistical analyses were performed using two-sided Wilcoxon rank-sum test. The exact sample sizes (*n*) are given as discrete numbers in fig. 3 and supplementary fig. S1, Supplementary Material online.

## Supplementary Material

msac195_Supplementary_Data

## Data Availability

The datasets generated during and/or analysed during this study are available from the corresponding authors on reasonable request.

## Supplementary material

Supplementary data are available at *Molecular Biology and Evolution* online. The datasets generated during and/or analysed during this study are available from the corresponding authors on reasonable request.
