# Supplementary material for: A CLAVATA3-like Gene Acts as a Gynoecium Suppression Function in White Campion
Source: Mol Biol Evol. 2022 Sep 27;39(10):msac195. doi: 10.1093/molbev/msac195 (PMC9550985; doi:10.1093/molbev/msac195)
Supplement: msac195_Supplementary_Data [file msac195_supplementary_data.pdf]

Supplemental Fig. 1

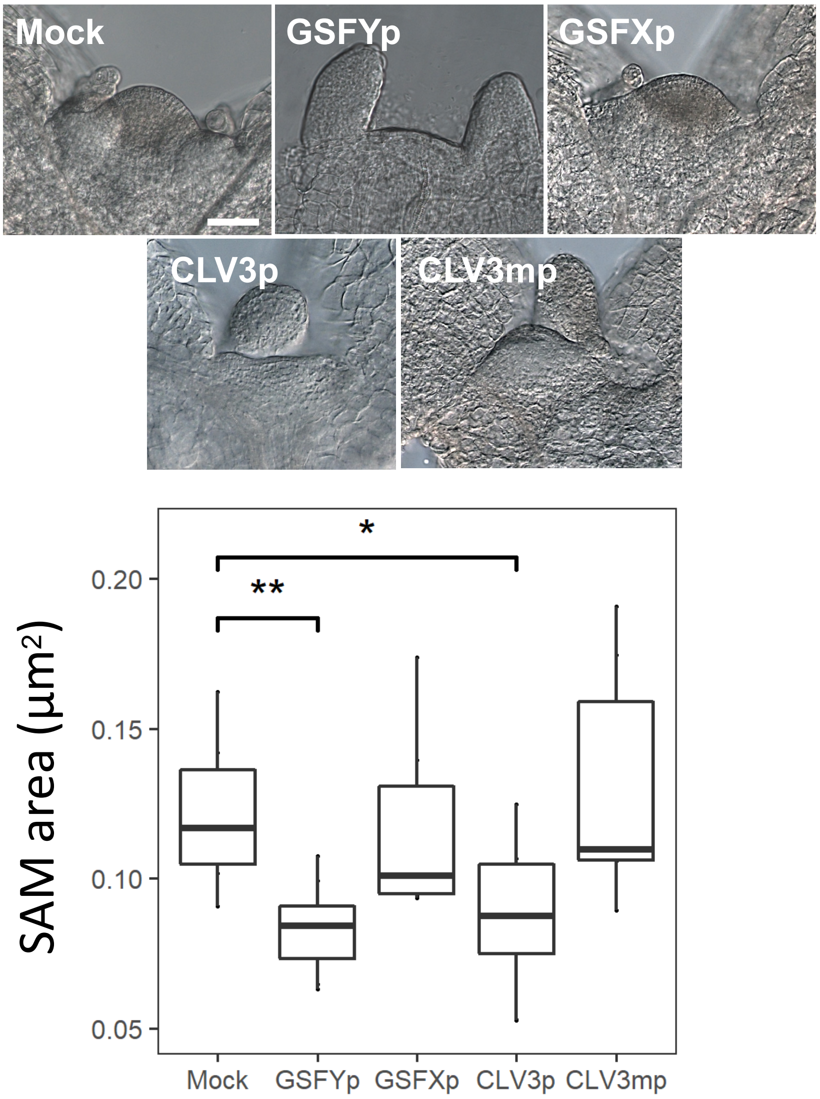

Fig. S1 The effects of different CLE peptides on *A. thaliana* SAM. CLE peptide treatment on the seedlings of clv3-101 *A. thaliana* mutants which is one of the null mutants of clv3. The SAMs were observed under a differential interference contrast (DIC) microscope after 5 d of growth on requid 1/2 MS media with 0.1 μM different peptides followed by calculation of SAM diameter by Image-J (at least four samples for each treatment). Upper panels shows photographs of representative plants. The lower panel shows quantitative measurements of the SAM area. GSFYp and CLV3p showed significant suppression of SAM growth ($p < 0.01$, and $p<0.05$, respectively), while GSFpX and CLV3mp showed almost no effect. Bar, 50 μm. Box plots, 25th-75th percentile; centre line, median; whiskers, full data range in B and D.

Supplemental Fig. 2

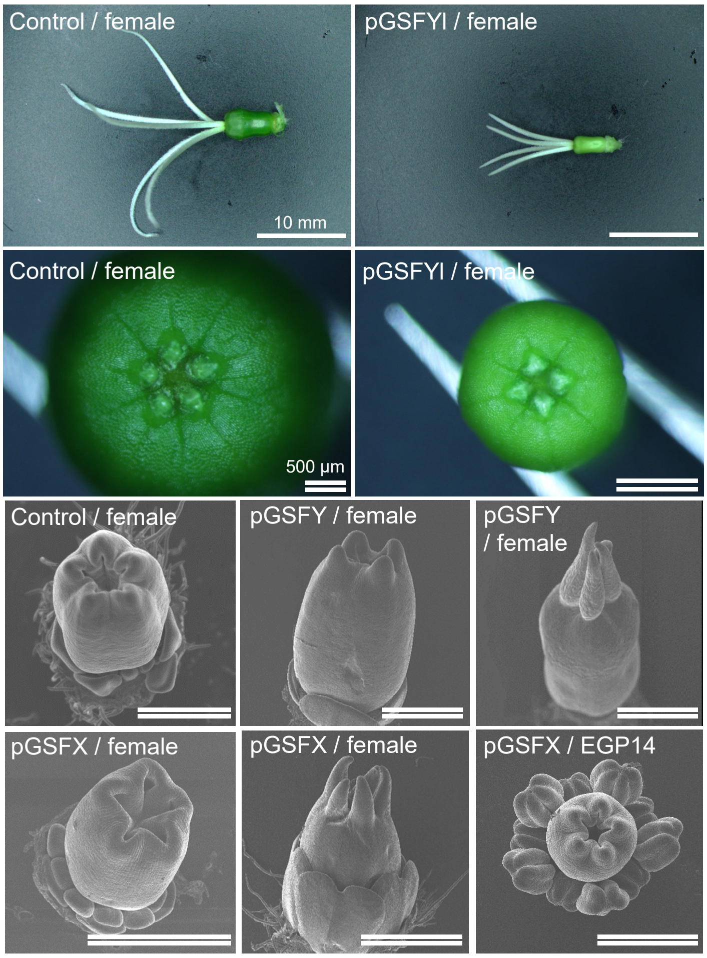

Fig. S2 The effect of GSFYp treatment on gynoecium development in *S. latifolia* female plants. Flower buds at stage 1-3 were treated with water containing 5 µM GSFYp. Open flowers were dissected and observed under stereo microscopy and SEM. GSFYp treatment decreased the size of ovaries and the number of styles, but neither females nor hermaphroditic mutant (EGP14) had decreased size of ovaries in the GSFXp treatment.

Table S1. Contigs identified identified by genomic seq and RNA-seq to narrow down candidate genes.

Contig ID	Sequence
C282650	CCTAATCTTGTTTGGTTCAACCCAAGTTATAGAAGGGGGGATTGAGTTTGAATCCAAG<br>GGGAGATGGTAGGTATGAGATTTCAAGGGGTTGAGGTGTAGTTAGAATCCATTTGGTA<br>TCTAACTTCATTCCTAAATTACCCAACCAAACACTTGAATGGACTCAATTAATTCCATTC<br>CATTCCAAAGTAGCCAACCAAGCACCCTTTAGTAGATTCCGGCCTAATCAGTAGATTGA<br>CCAAGCAATCTATTATTTTGATGTGTTTGGTAAACGACGTATTTTGACAATATATTGGC<br>CGAAATCTATTGTTTTTTAATATGCTCCTAATAGTAGCATATTGTATTAGCATATTCAAT<br>TTATACATGAAACCATTTTAATTATCCTATAATCTGCTAATTACCAAACACCCTTTTCTA<br>ATCCTGCTAATTTAATTTGATAGTCAAACCTGCTACTAAAAATTTACTAACGTTATCCGC<br>TATCTTGAATCTGTTTATGCTGTTTACCAAACAGGACCATACATATGTTAAGGTAAACA<br>ACATTTTGTAAAACAAGGTAATATGCTGTTTTAATATGTTATTATGTTGTTTACCAAATA<br>CCAAAGTTGACATAAGAGTGGCAAAAAAACTTTGAATATGCTAAAAGTCTTCCAAAATG<br>ATTTTTATTACCAAACATCTTCTATGTCATTGTATAATTGTACCAAATGTCTAATTATCTT<br>TTTAGCTTATTTATCCGATAATTTAAGTTGATTAATTAGCTATTTATATGTTTTTGCTATC<br>TTTTGAATTTCAGTTTCTTTGCCGTTTTCAGTTAACTTTTTAATTGATCTCACCAAACATA<br>TAAACATTGGGAAATTGATCTCACCAAACCTATAAACATTGGGAAAGAAAACCCTATAT<br>CTCTACATGAAATCATCTCACAAAAAACAATATTATGGAAAAATATCACTATCTCTACAA<br>GTCCACAAGTTGCCCATTAACATTAGAGCTAACAATATTATGATCGGTGGCGGAGTCA
C280774	CATTGTATTTTAAAAGCTAGTTCTGAGATTGAAACACACCGCAACTAAACAAAAGCACC<br>TAAGGGAATTTCCATCTAGCCCAGAGCATCGCCGTTTAAGTGCGTAGAACTCCTGTAAT<br>GCAAAAAAAAAAGATAATCTAAATAGTTAGTAGGCACAAGAAAACCAGATAAACTCTTC<br>AAGTTATCTATGTCATACAATTTAGTCACACTAAAAATGGATTGGGAACTGTCAAATAT<br>TTTCTTCTTTTGGTCGGGGAAGGGACACTGTCGGCATTAAAGAATCAAGTAGCTGGAA<br>TAAATAAATGTAAACCAAACTGACCTCTGCTTGACATCGCATATCAAAAGCATAGAGAA<br>CTCTATGAAAATATAGGGAGAAGCCGTTGTTTAGAAATGCTTCATAGTACATTCTGTTT<br>TTTTCATAATCTAAAGAAGCCGATTCACCCATCCCGATACTTAACATTTTATATAAATAG<br>ACTTCTGCAGCACCTAGTAATTCTACTCCCCAAACCTTGAGTAAAGTTTAAGGGAGTAT<br>TTTGTCAAACATCTGAACCTGCTCAGCCATATGTTTAACTCAACAGATGTATATGTATTT<br>AACACCCACACATTTTTTAGATTACACGATATTATTAAATCAATGAAGGAGCAAAAGGC<br>GACAAGGGGTTATAATGTAAAGATGCATAATTCCGTTGTATAAACATAATTGAGATACC<br>CTCTAAAAAACACCTGAAGCTCCCAAAATGATAATTTACAGTTGGTGCCATTCTGTTAG<br>TTTAATGCTAGGCATAGCTTTCATCCACCATACATGTTAAGTATAAGACTAAATCAGAA<br>TCTCAAGAACAAGTCAAACAATCTGTACAGAAGTAACAGAACTTCAAGTGAGAGCGTG<br>CTTTATAAACGGGTAA

Table S1. Continued

	ATCTTGAAAAACCTTAGTGTTCCAAGAAGCGTCACATATATATGTATTGATTCCAGGAT
	TTAAATTGATGAACCGGGGATATATTAATACTTTGTCAAAAATGAAAAAAGAAAAGTAA
	CAGCAACCGAAACTTCTTACTTCTGCTTTTACTTAATTCTACAACAGTCAACAGACCGT
	GTTCAGCTAAACAGCAAAGAACACAGTCATTCGAGATAACCCAAACCGGCTACGAAAA
	TAAACTTAAATTACATGACTTGAAAAAACGTAAACAAGCTAGCTAGGTTTTACTTTTGA
	ACTAACATTCAAAACAGACAGTGTTTTAGCTAAACAGCAAAAAAACACCGTTATTCGAG
	ATGACCAAACCGAAACTGGCTACGATAAACGTAAGTTTACATTTCTTGGAAAGGTAAAC
C279090	AACCAAGCTAGGTTTTACTTTTGAAGGTAACATTCAGAATCTGAGAAATAATCGTCAAG
	GACTAATTCTGGGCCTCTTTTGTGGGCCATTGTTGTGATGTACTGGATCTGGCCCAGAT
	GGTACATTCCTCGATTCCATATCATATTTGGACTTTGCCAGCTCCACTGCTCCTGATTTC
	TTTGTGCCCACTATCCCCATCAGAAACTGCAGGACATATTACACCTCAGTTAGGTTGTT
	CCCACTATTCAATACTTTGTATTACCTTGCTTATCCGTCTCGAACACTCGATAAGAATAG
	GACACTTATACATTTCATTCTAGACCAAAAGCATGAAATATTTATTCTGTATTCCGAATC
	AAAACGGTCAATGAAGTTATTTACTTGCCAATTTTACGAGAGTCTATTATAGTCGATAA
	GTCGAATCA

Table S2. Summary of sequence data generated for mutants and controls.

Individual	Data in Gb	Accession No.
**Genomic data**		
R025	273	DRR357638-DRR357639
Female	131	DRR360300
**Transcriptome sequence data**		
Male	4.20	DRR359968
R025	4.48	DRR359969
Male	23.3	DRR360400

Table S3. Primers used in this study.

Primer name	Sequence
C282650_F1	ATG GTA GGT ATG AGA TTT CAA GGG G
C282650_R1	TGA TTA GGC CGG AAT CTA CTA AAG G
C280774_F1	TGT CGG CAT TAA AGA ATC AAG TAG C
C280774_R1	CTT TAC ATT ATA ACC CCT TGT CGC C
C279090_F2	GCA AAG AAC ACA GTC ATT CGA GAT A
C279090_R2	CTT ACG TTT ATC GTA GCC AGT TTC G
GSFY_F7	ATG TCT ACT ACT TAT AAT AGT GTT ACT G
GSFX_F3	CGT CCC TTT TAA GCC AAC TG
GSFX_R2	CCG AAA TAA CCG AAA CTG A
SLactin01	TTA CCG TAA AGG TCC TTC CTG AT
SLactin02	AGC TTC GTG TTG CTC CTG AAG A
GSFY_cF2	ATC ATG GCA GAT TGT TAC GTC TCT T
GSFY_cR2	ATC TTC TAG AAA GAT TCA AGG ACT AAT TCT GGG CC
GSFY_gF2b	GTT TTT CAG CAA GAT CAC GTT TTC GAC AAA ATT GC
GSFY_gR2	ACA ATC TGC CAT GAT CAC AAG CAA AAT CAT CAG T
GSFY_gF1b	TAT ATG TTT GAA GCT CAC GTT TTC GAC AAA ATT GC
GSFY_gcR1b	GAT CGG GGA AAT TCG TCA AGG ACT AAT TCT GGG CC
GSFX_gF1	TAT ATG TTT GAA GCT GAA TTC GGA AGA TGA CAA TG
GSFX_gcR1	GAT CGG GGA AAT TCG TCA TGG ACT AAT TAT GGG CC
